# Posttranslational Amelogenin Processing and Changes in Matrix Assembly during Enamel Development

**DOI:** 10.3389/fphys.2017.00790

**Published:** 2017-10-17

**Authors:** Mirali Pandya, Tiffani Lin, Leo Li, Michael J. Allen, Tianquan Jin, Xianghong Luan, Thomas G. H. Diekwisch

**Affiliations:** ^1^Texas A&M Center for Craniofacial Research and Diagnosis, Dallas, TX, United States; ^2^UCLA School of Dentistry, Los Angeles, CA, United States; ^3^Brodie Laboratory for Craniofacial Genetics, University of Illinois at Chicago, Chicago, IL, United States; ^4^University of Michigan Medical School, Ann Arbor, MI, United States; ^5^Biometrology Inc., Chicago, IL, United States; ^6^Biocytogen, Worcester, MA, United States

**Keywords:** amelogenin, extracellular matrix, self-assembly, stippled materials, apatite crystal growth

## Abstract

The extracellular tooth enamel matrix is a unique, protein-rich environment that provides the structural basis for the growth of long and parallel oriented enamel crystals. Here we have conducted a series of *in vivo* and *in vitro* studies to characterize the changes in matrix shape and organization that take place during the transition from ameloblast intravesicular matrices to extracellular subunit compartments and pericrystalline sheath proteins, and correlated these changes with stages of amelogenin matrix protein posttranslational processing. Our transmission electron microscopic studies revealed a 2.5-fold difference in matrix subunit compartment dimensions between secretory vesicle and extracellular enamel protein matrix as well as conformational changes in matrix structure between vesicles, stippled materials, and pericrystalline matrix. Enamel crystal growth in organ culture demonstrated granular mineral deposits associated with the enamel matrix framework, dot-like mineral deposits along elongating initial enamel crystallites, and dramatic changes in enamel matrix configuration following the onset of enamel crystal formation. Atomic force micrographs provided evidence for the presence of both linear and hexagonal/ring-shaped full-length recombinant amelogenin protein assemblies on mica surfaces, while nickel-staining of the N-terminal amelogenin N92 His-tag revealed 20 nm diameter oval and globular amelogenin assemblies in N92 amelogenin matrices. Western blot analysis comparing loosely bound and mineral-associated protein fractions of developing porcine enamel organs, superficial and deep enamel layers demonstrated (i) a single, full-length amelogenin band in the enamel organ followed by 3 kDa cleavage upon entry into the enamel layer, (ii) a close association of 8–16 kDa C-terminal amelogenin cleavage products with the growing enamel apatite crystal surface, and (iii) a remaining pool of N-terminal amelogenin fragments loosely retained between the crystalline phases of the deep enamel layer. Together, our data establish a temporo-spatial correlation between amelogenin protein processing and the changes in enamel matrix configuration that take place during the transition from intracellular vesicle compartments to extracellular matrix assemblies and the formation of protein coats along elongating apatite crystal surfaces. In conclusion, our study suggests that enzymatic cleavage of the amelogenin enamel matrix protein plays a key role in the patterning of the organic matrix framework as it affects enamel apatite crystal growth and habit.

## Introduction

Tooth enamel is a remarkable bioceramic characterized by extraordinary hardness, resilience and fracture resistance. The formation of this extremely hard biomineral within the soft and gel-like extracellular enamel matrix remains an enigma in biomedical research to this day. In-depth understanding and visualization of the biological processes and mechanisms involved in amelogenesis are hampered by the limitations of conventional imaging techniques and artifacts introduced because of sample preparation. Specifically, optical *in vitro* and *in vivo* imaging are limited by the resolution of conventional light microscopy; scanning and transmission electron microscopy are limited by sample preparation for near-vacuum electron beam imaging conditions, contrasting procedures for organic matrix visualization, and beam damage to the mineral phase; atomic force microscopy provides high resolution but is restricted to surface topographies; and nuclear magnetic resonance spectroscopy lacks the topographical information provided by electro-optical imaging techniques. Yet, disregarding the weaknesses of individual strategies, the combination of information derived from complimentary approaches has yielded much progress toward a comprehensive understanding of the many aspects contributing to early enamel biomineralization. The combination of imaging and analytical techniques together with a plethora of individual approaches has resulted in a number of model systems that explain various aspects of enamel biomineralization.

Early enamel researchers thought of the enamel matrix as a “concentrated amorphous gel structure, rather than a more highly oriented assembly of fibers”(Fearnhead, 1963)and believed that the concept of the enamel matrix as a thixotropic gel would explain its “potential mobility as the protein would flow from regions where rapid growth of apatite crystallites caused a local increase in pressure to adjacent, relatively unmineralized regions, where it could initiate further crystals” (Eastoe, 1963). John E. Eastoe fathomed that the enamel proteins should be considered “as the true matrix of enamel in which the apatite crystallites are laid down, by a process which is not yet explored by which may be analogous to the epitactic mechanism believed to occur in collagenous mineralized tissues”(Glimcher, 1959; Eastoe, 1963). Thus, Eastoe imagined the enamel matrix as a homogeneous gel in which the enamel proteins freely float between further crystallized regions close to the dentin-enamel junction to the sites of early apatite crystallization at the ameloblast cell membrane, readily aiding each crystal to be initiated and grown “until they come into contact with their neighbors” (Eastoe, 1963). Notwithstanding this insightful speculation related to the function of the enamel matrix, Eastoe deserves much credit for the discovery of amelogenins as tissue-specific enamel proteins rich in proline, glutamic acid, and histidine (Eastoe, 1960).

Eastoe's contemporaries, the electron microscopists Dorothy F. Travis and Marie U. Nylen, pioneered an ultrastructural perspective of the developing enamel matrix by recognizing the stippled or finely granular materials located at the mineralization front as morphological building blocks of the enamel matrix (Frank et al., 1964; Travis and Glimcher, 1964; Reith, 1967; Nylen, 1979). They and others reported the presence of 5–7 nm granules in sectioned material and in suspensions of developing enamel (Fearnhead, 1965; Nylen, 1979). The existence of stippled materials was briefly called into question when the effect of fixative temperature on matrix structure was discovered (Lyaruu et al., 1982, 1984). Changes in enamel suprastructure at 4°C temperature had been described earlier and attributed to the high proline content of the enamel matrix (Nikiforuk and Simmons, 1965). Needless to say, faithful ultrastructural examination of the enamel matrix requires fixation at 37°C or room temperature as the mammalian body temperature does not drop to 4°C (Diekwisch et al., 1993, 1995). The functional significance of the enamel matrix stippled materials as supramolecular subunit compartments responsible for the control of enamel crystal growth became evident in study in which the translation of the key enamel matrix protein amelogenin was inhibited using an antisense strategy (Diekwisch et al., 1993). The concept of enamel matrix supramolecular assemblies as the basis for enamel crystal spacing and growth was thereafter confirmed using organ culture data (Diekwisch et al., 1995) and atomic force microcroscopy of unfixed freshly prepared enamel matrix (Diekwisch et al., 1993, 1995; Diekwisch, 1998).

Inversely interpreted transmission electron micrographs together with atomic force microscopy images and dynamic light scattering data helped to advance the nanosphere theory of enamel crystal growth (Fincham et al., 1994, 1995; Paine et al., 2001). A simplified sketch (Fincham et al., 1999) illustrates beaded rows of amelogenin nanospheres surrounding growing enamel crystals, and begs for the question as to how the needle-shaped thin enamel crystals would possibly grow while surrounded by densely packed globular structures. Indeed, the task of reconciling the rounded globes of amelogenin nanospheres with the sharp-edged hexagonal cross-sections of enamel apatite crystals resembles the fitting of a square peg in a round hole. Today, none of the three pillars of the nanosphere theory clearly provides evidence for the presence of spherical subunits in the enamel matrix: (i) light scattering data simply reference radii and not necessarily imply the presence of spherical assemblies, (ii) atomic force micrographs visualize the surface topographies of enamel proteins assembled on mica sheets and not in three dimensions, and (iii) the electron micrographs initially recruited to support the nanosphere theory incorrectly refer to the circular spaces in between nanospheres as protein assemblies instead of the electron dense protein coats associated with the growing enamel crystals (Diekwisch, 1998), a reversal of stained and unstained matrix compartments analogous to Edgar Rubin's young girl/old woman optical illusion. Moreover, the concept of self-assembly of amelogenins into spherical subunits is not universally accepted, as some investigators have argued that the organic enamel matrix organizes into fibrillar (Frank et al., 1960), lamellar (Ronnholm, 1962), or helical structures (Smales, 1975), or filaments and ribbons (Martinez-Avila et al., 2012; Carneiro et al., 2016). Recent small angle X-ray scattering (SAXS) studies propose that amelogenins self-assemble as nano-oblates with a 1:2 aspect ratio (Aichmayer et al., 2005; Margolis et al., 2006). Nevertheless, the nanosphere theory has established a model for the role of globular enamel protein assemblies as structural entities involved in enamel hydroxyapatite crystal growth.

The ubiquitous presence of the amelogenin-rich extracellular enamel matrix throughout all stages of enamel crystal formation infers an involvement in multiple aspects of matrix-mediated enamel crystal growth, including (i) matrix assembly, (ii) enamel crystal nucleation, (iii) initial crystal fusion of apatite precursors into apatite ribbons, and (iv) eventual crystal elongation and growth of true apatite crystals. Three models have been established to explain amelogenin nanosphere assembly and interaction among nanospheres. A first model based on SAXS data postulates that amelogenin nanospheres assemble into nanospheres with a dense hydrophobic core and a shell of hydrophilic and negatively charged chain segments (Aichmayer et al., 2005; Margolis et al., 2006). A second model, the amelogenin micelle model, focuses on the distribution of hydrophilic and hydrophobic regions within the amelogenin molecule and hypothesize that amelogenins aggregate into micelles through the ionic interactions between positively and negatively charged mini-domains and the complementary domain of another amelogenin molecule in reverse orientation (Fukae et al., 2007). A third model based on heteronuclear single quantum coherence nuclear magnetic resonance (HSQC NMR) spectra and analytical ultracentrifugation proposes that amelogenins self-assemble as donut-shaped entities through ipsilateral interactions at the α-helical N-terminus of the molecule, while the hydrophilic C-termini point toward the outside of the assembly (Zhang et al., 2011). Together, these studies provide a good understanding of the *in vitro* self-assembly capacity of amelogenin into nanoscale subunits. However, a universally accepted model explaining the *in vivo* structural entities of protein-mediated enamel crystal growth and their transformation throughout development is still lacking and in need of further investigation (Ruan and Moradian-Oldak, 2015).

Three recent *in vitro* studies have shed light on the possible protein/mineral interactions that take place during the onset of enamel crystal growth. The first of these three studies took advantage of a constant composition crystallization system, allowing for the control of ion concentration changes at the nanomolar level (Tomson and Nancollas, 1978). When used in combination with recombinant porcine amelogenin, this constant composition crystallization approach yielded hierarchically organized amelogenin and amorphous calcium phosphate (ACP) nanorod microstructures involving the coassembly of amelogenin-ACP particles (Yang et al., 2010). Second, a cryoelectron microscopy-based study has further confirmed that amelogenin undergoes stepwise hierarchical self-assembly, and that these assemblies are involved in the stabilization of mineral prenucleation clusters and their arrangement into linear chains (Fang et al., 2011). This study also demonstrated that the prenucleation clusters subsequently fused to form needle-shaped mineral particles and subsequently apatite crystallites (Fang et al., 2011). Finally, a combined circular dichroism/nuclear magnetic resonance (CD/NMR), dynamic light scattering, and fluorescence spectroscopy study resulted in a model for nanosphere formation via oligomers, suggesting that nanospheres disassemble to form oligomers in mildly acidic environment via histidine protonation (Bromley et al., 2011). In their model, amelogenins undergo stepwise self-assembly from monomers at pH3.5 to oligomers at pH5.5 and to nanospheres at pH8, while subsequent nanosphere breakdown would increase the amelogenin binding surface area to interact with the apatite crystal surface (Bromley et al., 2011). All three of these models postulate a very close interaction between the mineral and the protein phase at the site of initial calcium phosphate crystal growth. Such an intimate relationship between the organic protein matrix and the growing crystal phase goes back to earlier concepts proposed as part of Ermanno Bonucci's crystal ghost theory (Bonucci et al., 1988; Bonucci, 2014).

A number of morphological findings have helped to further expand our understanding of enamel crystal growth beyond the nanosphere stage, including the visualization of rows of globular assemblies on the surface of developing enamel hydroxyapatite crystal planes via freeze fracture electron microscopy (Moradian-Oldak and Goldberg, 2005), reports of nanosphere disassembly and “shedding” of amelogenins onto apatite surfaces and associated changes in amelogenin secondary structure (Tarasevich et al., 2009a,b, 2010; Lu et al., 2013), and the binding of globular matrix protein assemblies to developing enamel crystals *in vitro* (Robinson et al., 1981; Wallwork et al., 2001). Together, these findings lend support for a classic model of matrix mediated enamel crystal growth, henceforth dubbed the beehive model, which is comprised of strings of mineral/matrix nuclei that form a mantle of hexagonally arranged enamel mineral precursor deposits on the surface of growing enamel apatite crystals (Robinson et al., 1990).

Most recent reports about filamentous amelogenin nanoribbon self-assembly and their potential impact on enamel crystal formation add a unique dimension to the many shapes and forms resulting from amelogenin intermolecular associations (Martinez-Avila et al., 2011, 2012; Carneiro et al., 2016). Originally, these filamentous amelogenin nanoribbons were detected at water-oil interfaces (Martinez-Avila et al., 2011) or in the presence of calcium and phosphate ions (Martinez-Avila et al., 2011). Similarities between amelogenin nanoribbons and the amyloid polyglutamine fibrillar aggregates as they occur in neurodegenerative diseases (Chen et al., 2002; Tanaka et al., 2002; Schneider et al., 2011; Lyubchenko et al., 2012; Buchanan et al., 2014) have been invoked to explain the concept of nanoribbons templating apatite growth in human enamel (Carneiro et al., 2016). However, elongated enamel protein matrix ribbons without a close association to the adjacent mineral do not occur during enamel development *in vivo* (Diekwisch et al., 1995, 2002), and the protein assemblies generated in the filamentous nanoribbon studies are rather evidence of the unique propensity of amelogenins to form elongated assemblies *in vitro* than a physiological occurrence during mammalian amelogenesis. Nevertheless, this propensity of amelogenins to form elongated protein/mineral assemblies is likely a major force contributing to c-axis enamel crystal growth.

The present contribution seeks to introduce a developmental approach toward the relationship between enamel ions and proteins during enamel crystal formation and growth. Here we hypothesize that enamel ions and proteins are intimately associated with each other throughout the course of amelogenesis, starting from ion transport until advanced crystal growth, and that changes in mineral habit and protein conformation are caused by amelogenin enamel protein fragmentation. To verify our dynamic three-phase model of enamel matrix transformation and crystal growth (**Figure 4**) we have interrogated electron micrographs of developing mouse molar enamel *in vivo* and *in vitro* and analyzed amelogenin self-assemblies using atomic force microscopy, fluorescence microscopy, and nickel-labeling of the amelogenin N-terminus. To ask whether stage-specific changes in enamel matrix configuration were related to the presentation of amelogenin cleavage products within the matrix and adjacent to the crystal surface, we have separated porcine tooth molars into enamel organ, superficial and deep enamel preparations and performed a two-step protein extraction procedure separating loosely bound and mineral bound enamel proteins and probed protein extracts using N- and C-terminal amelogenin antibodies on Western blots. Together, these data provide new insights into the conformational changes of enamel matrix structure and related effects of amelogenin processing that take place during enamel matrix assembly, enamel crystal nucleation, and enamel crystal growth.

## Materials and methods

### Animal experiments and organ culture

For the preparation of 2 days postnatal mouse molars, mice were sacrificed according to UIC animal care regulation, molars were dissected from mandibles and immersed into Karnovsky's fixative as previously described (Diekwisch et al., 1995). For tooth organ culture studies, E16 timed-pregnant Swiss-Webster mice were sacrificed and mandibular first molars were dissected.

El6 cap stage tooth organs were cultured for 12 days in BGJb+ medium (Fitton-Jackson's modified BGJ medium) supplemented with 100 g/ml L-ascorbic acid and 100 U/ml penicillin/streptomycin as previously described (Diekwisch et al., 1995). Explanted molars were cultured at 37°C with 95% air and 5% CO_2_. Initial pH was adjusted to 7.4 and the medium was changed every other day.

### Transmission electron microscopy

Three days postnatal mouse molar tooth organs as well as E16 tooth organs cultured for 12 days were fixed in Karnovsky's fixative as previously described (Diekwisch, 1998), dehydrated and embedded in Eponate 12 (Ted Pella, Redding, CA). Sections were cut on a Leica Ultracut UCT ultramicrotome. After drying, sections were contrasted in 1% uranyl acetate followed by Reynold's lead citrate for 15 min each. Observations were made on a JEOL 1220EX transmission electron microscope at the UIC Research Resources Center (Chicago, IL).

### Proteins

The full length mouse amelogenin (M179), the N-terminal amelogenin N92 coding sequence, and the C-terminal amelogenin C86 were cloned into pASK-43(+) with EcoR I and XhoI restriction sites at the 5' and 3' end respectively as previously described(Jin et al., 2009; Zhang et al., 2011). M179 is the full-length mouse amelogenin protein lacking the N-terminal methionine (Simmer et al., 1994), while the terms N92 and C86 denote recombinant proteins based on the N-terminal amelogenin 92 amino acid fragment or the C-terminal amelogenin 86 amino acid fragment (Zhang et al., 2011). For nickel staining of the N-terminal polyhistidine tag, an N-terminal MRGSHHHHHHGAGDRGPE HIS-tag was inserted at N-terminus of the protein. BL21-DM^*^ host bacteria were cultured at 37°C until the OD_600_ reached 0.8 and then were induced at 32°C for 4 h. The expressed proteins were absorbed onto a Ni-NTA agarose column and washed with 10 column volumes of PBS and 3 column volumes of 40 mM imidazole in PBS, followed by protein elution with a pH 5.0 gradient (from 50 to 500 mM) imidazole PBS solution and dialysis against H_2_O. Subsequently, the purified proteins were concentrated to about 10 mg/ml using a Centriprep YM-3 column. Finally, the polyproline repeat amelogenin PXX33 peptide (>99% purity, sequence PMQPQPPVHPMQPLPPQPPLPPMFPMQPLPPML) was synthesized by Genescript (Piscataway, NJ).

### Atomic force microscopy

The atomic force microscope (AFM) measurements were carried out using an extended MultiMode AFM (MMAFM) integrated with a NanoScope IIIa controller (Veeco Instruments, Santa Barbara, CA) and a Q-Control Module (nanoAnalytics, Muenster, Germany) as previously described (Jin et al., 2009). The MMAFM was equipped with a calibrated E-type piezoelectric scanner and a glass cell for fluid TappingMode AFM (both from Veeco). The silicon AFM cantilever/probe used in this study was rectangular in shape, 130 μm in length and 35 μm in width (NSC36, MikroMasch). The advertised typical force constant and resonant frequency of this cantilever/probe is 0.6 N/m and 75 kHz respectively. Nominal sharpness of the probe-tip end radius is ≤10 nm. The cantilever/probes were oscillated near 30 kHz at low amplitude for fluid tapping mode AFM. Fluid damping reduces the resonant frequency of rectangular AFM cantilevers in air by approximately 50%. The AFM substrate used for protein adsorption was Grade V5, Pelco mica (10 × 40 mm) purchased from Ted Pella (Redding, CA). The mica was freshly cleaved using adhesive tape prior to use. Stock solutions of 10–20 mg/ml protein (either amelogenin M179 or C86) in 40 mM Tris (pH 8.0) were mixed and stored at 4°C and analyzed by AFM. Stock solutions were diluted typically at 1:100 into the blank AFM imaging buffer (40 mM Tris, pH 8.0) during scanning and adsorption to mica was monitored. Typical AFM scan rates were 1.0–1.25 Hz for 512 data points × 256 lines. The AFM images were planefit to correct for background sloping errors.

### Fluorescent images of aqueous protein assemblies

Lyophilized recombinant M179 full-length mouse amelogenin and synthesized PXX polyproline repeat peptide were immersed in DDW (pH 7.4) overnight and allowed to self-assemble on a glass slide kept within a humid chamber. Same amounts of each protein were used in this study. After 24 h, 1% fluorescein was added to the aqueous solution for 1 h. Subsequently protein solutions on glass slides were examined under a cover slip using a Leica fluorescent microscope with a 100x oil immersion lens.

### Polyhistidine tag labeling and electron microscopy

Droplets containing 100 μl of diluted (1 mg/ml) pH7.5–8.0 His-tagged recombinant N92 amelogenin were placed on carbon coated copper TEM grids (Ted Pella, Redding, CA) and incubated in a moisturized container at 37°C for 2 h. Thereafter, TEM grids were quickly rinsed with DDW, immersed into 100 μl of freshly prepared 1% NiSO_4_ (Sigma, St. Louis, MO) solution for 30 min, quickly rinsed with DDW again, air dried, and analyzed using a JEOL 1220EX transmission electron microscope at the UIC Research Resources Center (Chicago, IL).

### Western blot

Three months old porcine mandibles were obtained from a local animal farm, and enamel organ epithelium and enamel matrix proteins were collected immediately after slaughter from unerupted mandibular molars. As a first step, the epithelial enamel organ (EO) was collected separately from the matrix and subjected to protein extraction. As a second step, two successive layers of the protein rich enamel matrix were scraped off the tooth surface: (i) a superficial enamel matrix layer that was soft in consistency and easily removable without application of force (SEL), and (ii) a deeper enamel matrix layer that was already hardened and required mechanical force to be separated from the underlying and already mineralized dentin surface (DEL). Tissue and matrix from all three groups were then subjected to protein extraction for 5 days with SDS lysis buffer containing 0.5% sodium dodecyl sulfate, 0.05 M TRIS-Cl, 1 mMol dithiothreitol (DTT) with a pH of 8.0. After lysis, samples were dialyzed for 1 week at 4°C against DDH_2_O, and centrifuged for 15 min at 2,400 g and 4°C. As a first step, the SDS soluble supernatant from all three groups was collected for Western blot. After removal of the supernatant, the pellet of all three extracts was subject to a second round of extraction with 4 M guanidine HCl. After 5 days of extraction in 4 M guanidine HCL, the extraction solution was once more centrifuged, and the supernatant of the 4 M guanidine group of each group was collected and dialyzed for 1 week at 4°C. Thereafter, proteins were concentrated using Amicon spin columns (3 kDa cut-off, Millipore, Billerica, MA), and re-suspended in RIPA buffer for Western blot detection.

For Western blot analysis, equal amounts of protein were loaded onto a 10% SDS polyacrylamide gel, subjected to SDS gel electrophoresis and then transferred onto a polyvinylidine difluoride (PVDF) membrane using a semi dry transfer system. The membrane was blocked with 5% dry milk in TBST, probed either with primary antibody against the C-terminal amelogenin fragment or against the N-terminal amelogenin fragment (1:1,000), followed by anti-rabbit IgG HRP conjugated secondary antibody (1:2,000; cell signaling) incubation. Primary antibodies were based on the following amelogenin-derived peptides: LPPHP GSPGY INLSY EVLTP LKWYQ SMIRQ P (N-terminal antibody) and PLSPI LPELP LEAWP ATDKT KREEV D (C-terminal antibody) and generated in collaboration with Zymed (South San Francisco, CA). A chemiluminescent substrate (Thermo Scientific) was used to reveal the HRP signal.

### Statistical analysis

For this analysis, 15 subunit compartments located either in secretory vesicles or within the enamel matrix were selected using a random generator and average subunit size and standard deviations were calculated and reported for both groups. Student's *t*-test was used to determine statistically significant differences between the two groups and the significance level was set at α ≤ 0.05.

## Results

### Changes in matrix subunit compartment dimensions between secretory vesicle matrix, extracellular enamel protein matrix (“nanospheres”), and pericrystalline protein matrix (“crystal ghosts”)

**Figure 1:**
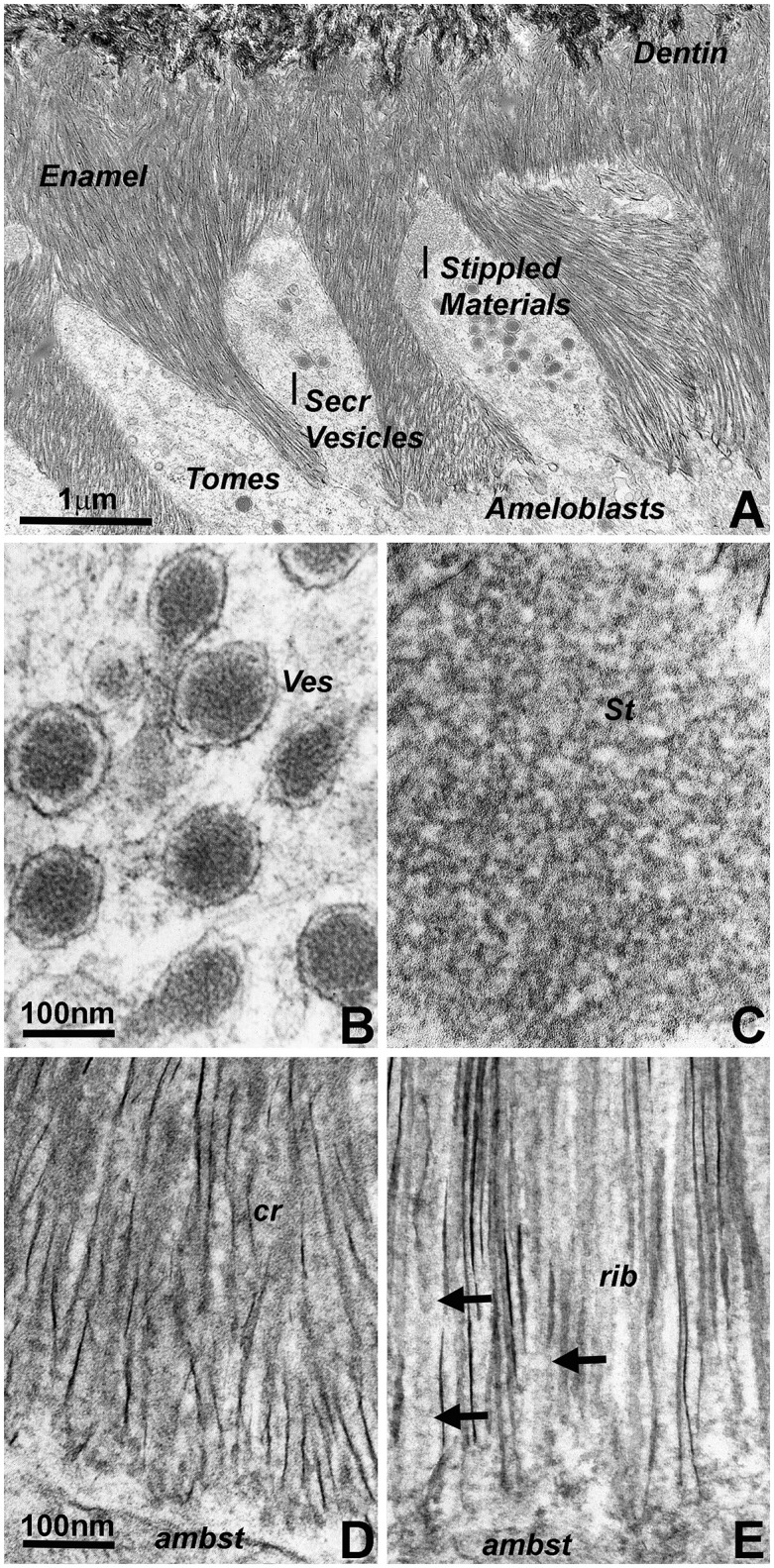
Electron micrographs illustrating subunit assembly and structural transformation during initial enamel crystal formation *in vivo*. **(A)** Interface between Tomes' processes and early enamel prisms. Note the presence of enamel matrix carrying secretory vesicles (Secr Vesicles) within the Tomes' process (Tomes) at the apical ameloblast pole. Bulk deposits of an extracellular matrix containing stippled materials were recognized between the ameloblast cell membrane and the newly formed enamel crystal layer. The border between enamel (Enamel) and dentin (Dentin) was delineated by differences in crystal structure and organization). **(B)** High resolution ultrastructure of an ameloblast secretory vesicle (ves). **(C)** Ultrastructure and subunit organization of the non-mineralized enamel extracellular matrix commonly identified as stippled materials (St). **(D,E)** Ultramicrographs of early enamel crystals. **(D)** illustrates the somewhat disorganized arrangement of initial enamel crystals (cr), and **(E)** reveals ribbon-shaped assemblies (rib, arrows) of organic matter in between highly parallel rows of enamel crystals. Scale bar **(A)** = 1 μm; **(B,C)** = 100 nm; **(D,E)** = 100 nm. The same scale bar applies for **(B–E)**.

Transmission electron micrographs of developing mouse molar enamel revealed three stages involved in matrix mediated enamel crystal growth (Figure 1A): (i) initial matrix assembly in ameloblast secretory vesicles, (ii) deposition of an extracellular enamel matrix consisting of stippled materials, and (iii) formation of initial enamel crystallites within this extracellular matrix. Comparison between Figures 1B,C illustrates the remarkable subunit size differences between the enamel matrix of the stippled materials (Figure 1C) and the matrix within the secretory vesicles (Figure 1B). Subunit dimensions were 7.07 nm ± 1.61 nm for the secretory vesicle matrix and 17.47 nm ± 3.44 nm in the extracellular enamel matrix of the stippled materials (Figure 1B vs. Figure 1C). The 2.5-fold difference in subunit size was statistically highly significant (*p* < 0.0001). Transmission electron micrographs also demonstrated the less than parallel alignment of the earliest enamel crystallites (Figure 1D) in comparison to the fairly parallel aligned crystal needles at a further advanced state of crystal growth (Figure 1E). In terms of matrix assembly, these images revealed electron dense globular organic enamel matrix subunits closely associated with growing enamel crystallites (Figure 1D) and beaded or helical arrangement of organic nanoribbons in close proximity to the elongating apatite crystals (Figure 1E). Exposure of isolated and free-standing enamel protein nanoribbons (arrows) was likely due to the thin plane of section on these 400Å diameter ultrathin sections (Figure 1).

### Key features of enamel crystal growth in organ culture: (i) granular mineral deposits associated with the enamel matrix framework, (ii) dot-like mineral deposits along elongating initial enamel crystallites, and (iii) “crystal ghost” organic matrix adjacent to forming enamel crystals

**Figure 2:**
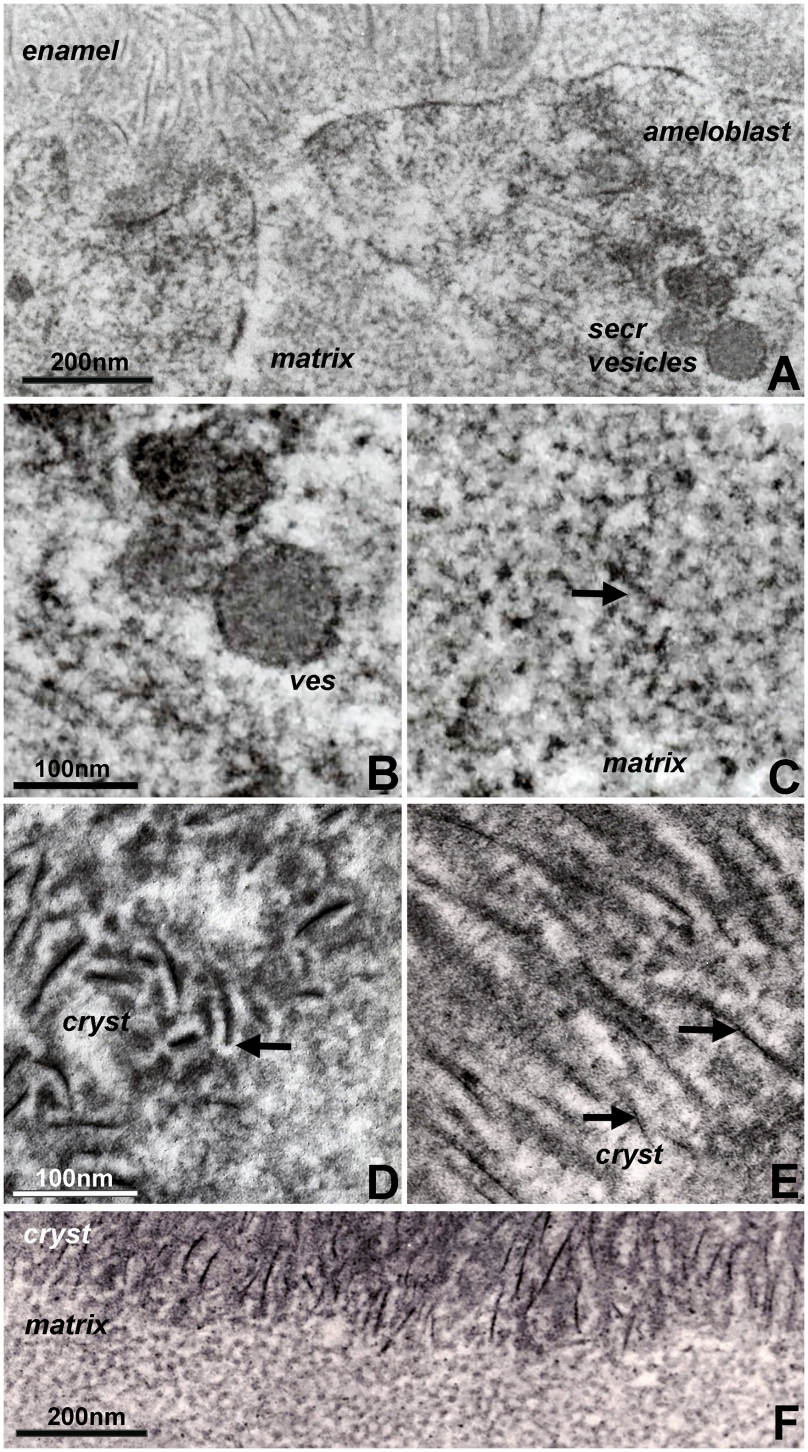
Constituents of first mandibular mouse molar enamel formation in organ culture as revealed by electron microscopy. Note the differences between Figure 1 (*in vivo*) and this figure (*in vitro*). **(A)** Interface between apical ameloblast cell membrane (ameloblast), organic extracellular enamel matrix (matrix), and initial enamel crystal deposits (enamel). Note the secretory vesicles (secr vesicles) at the apical ameloblast pole. **(B,C)** High magnification ultrastructural comparison between enamel matrix structure within secretory vesicles **(B)** and extracellular matrix **(C)**. The arrow in **(C)** points to electron dense mineral deposits as part of the supramolecular matrix framework. **(D,E)** Initial stages of enamel crystal (cryst) formation in organ culture. Note the electron opaque coat (arrow) surrounding initial crystal precipitates **(D)** and the electron dense particles (arrow) in immediate proximity of the elongated crystals **(E)**. **(F)** Sharply delineated interface between non-crystallized organic matrix (matrix) and the initial crystallized enamel layer (cryst). Scale bar **(A)** = 200 nm; **(B,C)** = 100 nm; **(D,E)** = 100 nm; **(F)** = 200 nm. The same scale bar applies for **(B–E)**.

Organ culture models are unique experimental systems in which the loss of circulation and the reduced access to nutrients allows for enhanced morphological insights into key events of mineralized tissue formation (Diekwisch et al., 1993, 1995; Diekwisch, 1998). Here, our tooth organ culture study revealed granular electron dense mineral deposits onto the organic matrix framework of the enamel matrix stippled materials (Figure 2C), suggestive of a high mineral content in the pre-crystalline enamel extracellular matrix. Initial crystallites were surrounded by a fairly electron dense organic matrix (Figure 2D). These initial mineral protein/mineral assemblies were separated from each other by electron-lucent zones in between discrete mineral assembly deposits (Figure 2D). Elongated crystals were surrounded by an electron dense coat of mineral granules in immediate proximity to the crystal surface, indicative of epitaxial crystal growth (Figure 2E). Finally, transmission electron micrographs of the enamel matrix/initial crystallization interface demonstrated an almost linear separation between the subunit compartments of the non-mineralized matrix and the crystal-associated matrix of the early crystalline phase, suggestive of an *en block* conversion of matrix assemblies from crystal-free to crystal-rich matrix (Figure 2F).

### Linear and 20 nm hexagonal/ring-shaped amelogenin protein assemblies on mica surfaces and 20 nm globular amelogenin assemblies of nickel-stained N92 amelogenins on carbon coated grids as revealed via AFM and TEM

**Figure 3:**
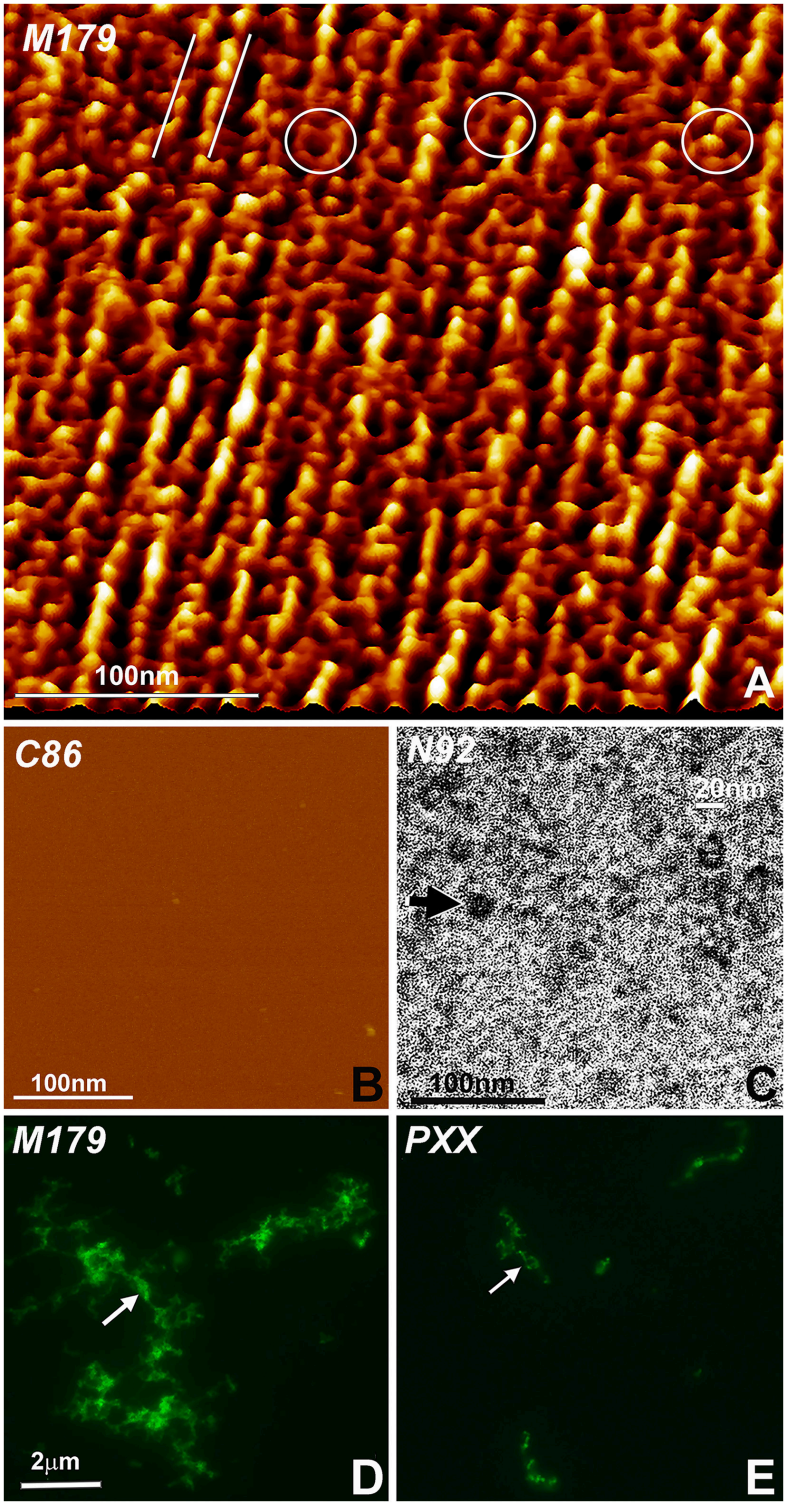
Amelogenin protein self-assembly *in vitro***. (A)** AFM image of M179 amelogenin on freshly cut mica. Note the parallel rows of self-assembled amelogenin spheres (parallel white lines) next to hexagon-shaped, ring-like assemblies (white circles). **(B)** In contrast, C86 amelogenin on freshly cut mica did not reveal any prominent structural entities. **(C)** Oval shaped, N-terminal His-tagged amelogenin N92 assemblies (arrow) as revealed by nickel-stain. **(D)** Fluorescently labeled, self-assembled full-length amelogenins in aqueous solution. **(E)** Fluorescently labeled, self-assembled amelogenin PXX33 polyproline repeat peptides in aqueous solution. The arrow points to elongated amelogenin structures **(D,E)**. The same scale bar applies for **(D,E)**.

Three different types of experiments were conducted to visualize modes of amelogenin self-assembly and address the question as to which amelogenin motifs were involved in self-assembly and protein elongation. In a first set of experiments, recombinant full-length mouse amelogenin (M179) and C-terminal C86 amelogenin were placed on freshly cleaved mica and allowed to self-assemble (Figures 3A,B). Tapping mode AFM images revealed parallel rows of globular amelogenin protein as well as circular/hexagonal inter-row assemblies (Figure 3A) indicative of a propensity of full-length amelogenins to self-assemble either in linear rows or as hexagonal patterned subunit compartments when exposed to flat mica surfaces at pH 7.4 without the addition of additional proteins or ions. The C-terminal amelogenin alone without the helical N-terminus did not form any detectable surface patterns (Figure 3B). To ask whether the amelogenin N-terminus was involved in self-assemblies, our previously generated N-terminally His-tagged N92 amelogenin (Zhang et al., 2011) was incubated on carbon-coated mesh wire grids and subjected to nickel staining. Transmission electron micrographs of stained N92 matrices revealed oval or donut-shaped electron-dense assemblies measuring approximately 20 nm in diameter (Figure 3C). Fluorescent labeling of overnight incubated amelogenins in aqueous solution at pH 7.4 resulted in complex large scale assemblies measuring several micrometers in length (Figure 3D). In contrast, self-assemblies of PXX33 polyproline-rich amelogenin peptides incubated under the same conditions were substantially thinner and smaller (Figure 3E).

### Western blot analysis reveals parallels between amelogenin fragmentation and changes in matrix organization during enamel protein transport, “nanosphere” assembly, and crystal growth

**Figure 4:**
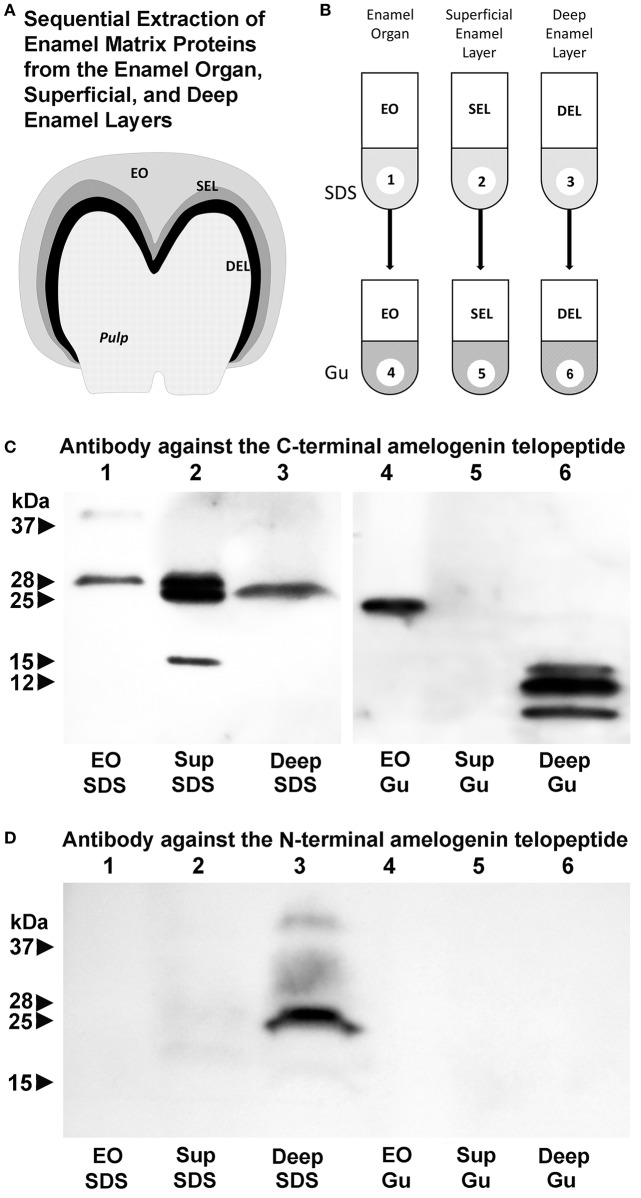
Localization of amelogenin fragments in enamel organ, superficial, and deep enamel layers based on fractionated protein extraction and antibody recognition site. **(A)** Porcine enamel organ dissection and preparation scheme. Enamel organ (EO), superficial enamel layer (SEL), and deep enamel layer (DEL) were separately collected for further analysis. **(B)** Fractionated enamel protein extraction procedure using sodium dodecylsulfate (SDS) as a first step and 4 M guanidine HCl for subsequent extraction of the SDS-insoluble residue as a second step. **(C)** C-terminal amelogenin peptide antibody based Western blots of SDS extracts (SDS) or SDS residue guanidine extracts (Gu) of EO, SEL, and DEL enamel organ/enamel layer protein preparations. **(D)** N-terminal amelogenin peptide antibody based Western blot of SDS extracts (SDS) or SDS residue guanidine extracts (Gu) of EO, SEL, and DEL enamel organ/enamel layer protein preparations.

Here we asked whether changes in enamel matrix configuration as they occur during amelogenesis coincide with the gradual processing of the full-length amelogenin into enzymatically cleaved fragments. In addition, we employed two successive stages of protein extraction to separate loosely-bound and crystal-associated matrix proteins. First, loosely bound intercrystalline proteins were harvested using a sodium dodecyl sulfate (SDS)-based extraction procedure that functions similar to a detergent. Thereafter, crystal-bound enamel matrix proteins were extracted via 4 M guanidine (modified after Termine et al., 1980). Individual SDS-based or guanidine (Gu)-based extracts from enamel organ, superficial or deep enamel matrix were then subjected to gel electrophoresis and Western blot (Figure 4A). We postulated that our layer- and binding-level based analysis would provide new insights into relationship between amelogenin processing, matrix assembly, and protein-mediated crystal growth.

Our C-terminal amelogenin antibody recognized a distinct 28 kDa band indicative of the full-length amelogenin on the SDS-based enamel organ extract (Figure 4C, lane 1). This antibody identified two strong bands at 28 and 25 kDa and a less intense band at 15 kDa on the SDS-based extract of the superficial enamel matrix, while there was a single 26 kDa band on the SDS-based extract of the deep enamel matrix (Figure 4C, lanes 2 and 3). There was a 23 kDa amelogenin positive band on the Gu-based extract of the enamel organ and a series of three amelogenin positive bands ranging from 8 to 16 kDa on the Gu-based extract of the deep enamel matrix (Figure 4C, lanes 4 and 6). In opposite to the strong amelogenin signal in the SDS extract of the superficial enamel layer, the amelogenin signal in the Gu extract of the superficial enamel was below detection threshold (Figure 4C, lane 5). In contrast, our N-terminal amelogenin antibody only reacted with the SDS-based extract of the deep enamel layer (Figure 4D), indicating that the N-terminal amelogenin fragment is not immediately associated with the growing crystal surface.

## Discussion

For the present contribution we have queried the developing enamel matrix using *in vivo* and *in vitro* models as well as amelogenin self-assembly patterns to reconcile seemingly divergent models and proposed mechanisms of mammalian matrix mediated tooth enamel formation. We have revisited electron micrographs of mouse enamel development, carefully analyzed lesser known aspects of enamel matrix reconfiguration and initial crystal growth in organ culture, and characterized amelogenin *in vitro* self-assembly patterns using atomic force microscopy, fluorescence microscopy, and nickel-labeling of the N-terminal polyhistidine tags at the N-terminus of amelogenin N92 fragments. We have also performed Western blot analyses to determine whether stage-specific changes in enamel matrix configuration were related to the amelogenin posttranslational processing along stages and layers of enamel development using N- and C-terminal amelogenin antibodies. Together, these studies establish the enamel matrix as a dynamic and multifunctional protein assembly involved in all aspects of enamel formation, including vesicular transport, matrix assembly, spacing of crystal nucleation sites, and protein mediated crystal elongation.

Our micrographs indicate substantial differences in matrix subunit dimensions and shapes between secretory vesicles, pre-crystallization enamel protein matrix, and intercrystalline protein matrix during the crystal elongation phase. Specifically, there was a significant difference in matrix subunit compartment size between secretory vesicle assemblies measuring approximately 7 nm in diameter and the extracellular enamel protein matrix subunit compartments with an average diameter of 17.5 nm. Similar changes in subunit dimensions have been reported in earlier molecular cross-linking studies (Brookes et al., 2006). A detailed analysis of matrix dimensions in an earlier transmission electron microscopic study reported 5 nm diameters in secretory vesicles and 20 nm diameters in stippled materials and in the protein coat covering initial enamel crystal deposits (Diekwisch et al., 1995). Estimates of protein assembly dimensions based on transmission electron micrographs are likely to underestimate actual dimensions by a small percentage because of the dehydration involved in sample preparation, suggesting that actual subunit dimensions may be closer to 10 nm in secretory vesicles and 25 nm in the extracellular matrix. Together, these findings indicate that the enamel matrix is reconfigured when the enamel mineral/protein cargo leaves the secretory vesicles and enters the extracellular matrix milieu. Our data are also suggestive of a second change in matrix configuration after initial crystal precipitation. In fact, the structures presented in our transmission electron micrographs somewhat resemble helical structures (Smales, 1975), but more likely consist of ribbon-like assemblies of donut-shaped protein nanospheres (Zhang et al., 2011; Carneiro et al., 2016) in immediate proximity to the elongating crystal needles. Such protein nanoribbons not always display the corresponding crystal needle in the same section because of the ultrathin sectioning technique involved in sample preparation. However, electron micrographs of earlier and later stages illustrate the intimate relationship between each individual electrondense enamel crystal needle and its slightly less electrondense pericrystalline protein coat. Similar images of pre-fusion initial enamel crystals and consecutive stages of apatite fusion into mature enamel crystals have been published earlier (Robinson, 2007; Beniash et al., 2009; Fang et al., 2011).

Organ culture studies revealed four key findings related to our understanding of potential mechanisms involved in enamel crystal growth: (i) granular mineral deposits associated with the enamel matrix framework, (ii) dot-like mineral deposits along elongating initial enamel crystallites, (iii) a mineral free-zone surrounding initial enamel crystal precipitates, and (iv) dramatic changes in enamel matrix configuration following the onset of enamel crystal formation. Organ cultures are unique experimental environments that faithfully mimic the timely progression of physiologic events during embryonic organogenesis (Trowell, 1954; Yamada et al., 1980; Saxen et al., 1983; Evans et al., 1988). However, because of a limited supply in nutrients, limited ion and protein diffusion, isolation from surrounding tissues, and physical separation from long-range signaling events, amelogenesis in organ culture is effectively a time-lapse process that progresses at approximately twice the speed of *in vivo* amelogenesis. The time-lapse progression of events and the slight augmentation of key morphological features due to an accumulation of matrix and mineral allows for the visualization of events and structures that would otherwise remain below the threshold of detection (Diekwisch et al., 1993, 1995; Diekwisch, 1998).

Among the unique findings presented here is the evidence for granular mineral deposits along the stippled materials framework of matrix subunit compartments, suggesting that the stippled materials structure previously thought of as a mineral-free protein zone in fact contains a mixture of mineral ions and proteins. This finding and the detection of dot-like, granular mineral deposits along the elongating apatite crystal surface not only confirm earlier reports of linearly arranged, electron-dense dots and globular subunits (Frank and Nalbandian, 1963; Hohling et al., 1966; Robinson et al., 1981, 1983), but also lends support to more recent concepts involving co-assembled amelogenin protein/calcium phosphate mineral nanoclusters as the basis for enamel mineral growth (Beniash et al., 2005, 2009; Yang et al., 2010; Bromley et al., 2011; Ruan and Moradian-Oldak, 2015). In fact, the presence of an electron lucent zone surrounding initial crystal precipitates with adjacent matrix deposits in organ culture may indicate that protein/mineral nanoclusters had disassembled (“shed”) from nanospherical matrix subunits onto the crystal surface and were no longer present at the interface between crystals. One of the most remarkable sights in our electron micrographs of initial enamel mineralization *in vitro* and *in vivo* was the drastic conversion of matrix structure from the stippled materials matrix to the elongated protein and mineral assemblies of initial crystal growth. Such a conversion of matrix organization may be due to the deprotonation of amelogenin histidine residues and simultaneous protonation of crystal surfaces, resulting in the disassembly and shedding of nanosphere substructures (Tarasevich et al., 2009a,b; Bromley et al., 2011; Robinson, 2014; Ruan and Moradian-Oldak, 2015), and the initiation of a cascade of events related to crystal formation, epitaxial crystal growth, and crystal elongation.

Our atomic force micrographs of full-length amelogenin *in vitro* self-assemblies on freshly cleaved mica not only demonstrate that amelogenins have the capacity to form linear protein assemblies but also self-organize into hexagonal rings resembling the subunit compartment organization of the stippled enamel extracellular matrix. As striking as those linear protein assemblies might be, careful examination of these images reveals the large number of hexagonal ring subunits in between rows of globular protein structures. As mentioned earlier, the linear arrangement of protein subunits may be evidence of the unique propensity of amelogenins to form elongated assemblies, which in turn might facilitate longitudinal enamel crystal growth along the crystal c-axis. As to the involvement of individual amelogenin motifs in amelogenin self-assembly, our nickel labeling of the N92 amelogenin polyhistidine tag confirms the essential role of the amelogenin N-terminus in the self-assembly of 20 nm diameter aggregates (Zhang et al., 2011). In contrast, our fluorescein labeling studies indicate that the polyproline domain alone results in very limited protein self-assembly and might rather contribute to nanosphere compaction and enamel prism formation (Jin et al., 2009), while the C-terminus has been shown to preferentially bind to the (100) face of apatite crystals when compared to the (001) phase and contribute to c-axis crystal growth (Moradian-Oldak et al., 2002; Pugach et al., 2010; Friddle et al., 2011; Gopinathan et al., 2014).

Our Western blot analysis of sequentially extracted enamel matrix proteins from the enamel organ, superficial and deep enamel matrix layers revealed a 3 kDa cleavage of the full-length amelogenin when the protein leaves the enamel epithelium, enters the enamel matrix, and then associates with the crystal surfaces. This finding indicates that the amelogenins of the enamel organ epithelium are of higher molecular weight than the amelogenins in the enamel matrix. Such higher molecular weight (28 kDa) amelogenins likely provide the structural framework for the 5–8 nm subunit assemblies within the ameloblast secretory vesicles. Once expelled from the ameloblast cell body and upon entry into the enamel matrix, the transition from ameloblast secretory vesicle subunit compartments into 20 nm enamel matrix “nanosphere” assemblies is likely accomplished by C-terminal amelogenin cleavage via the matrix metalloproteinase MMP20 (Zhu et al., 2014) into slightly lower molecular weight (25 kDa) amelogenins. MMP20 is abundant at the ameloblast/enamel matrix interface and activated in the proton-rich environment of initial apatite crystal formation (Khan et al., 2012). The C-terminal cleavage then results in a reassembly of the enamel protein matrix structure from the 5–8 nm subunit assemblies into the 20 nm matrix subunit compartments.

The second key finding of our Western blot analysis focuses on the transition from the loosely bound and SDS extractable 25/28 kDa amelogenins of the superficial enamel matrix to the crystal associated 8–16 kDa C-terminal amelogenin fragments that were only resolved after subsequent guanidine extraction. In our laboratory, 4 M guanidine alone is commonly employed to cause a profound dissolution of the mineral phase, even though addition of EDTA would result in further removal of the enamel mineral. Changes in amelogenin molecular weight from the full-length molecule in the superficial enamel layer to shorter fragments in the crystal-bound phase explains the dramatic change in enamel matrix configuration from “nanosphere”-type supramolecular matrix assemblies to the “crystal ghost”-type organic crystal coverings on the surface of elongating apatite crystals as a result of further enzymatic processing. This finding confirms previous studies on the close proximity of the amelogenin C-terminus to the apatite surface (Tarasevich et al., 2009a,b, 2010; Lu et al., 2013). In contrast to the apatite-associated amelogenin C-terminus, the amelogenin N-terminus was accessible to our SDS solvent based extraction procedure, suggesting that the N-terminal amelogenin resided loosely bound in the intercrystalline space of the deep enamel layer.

**Figure 5:**
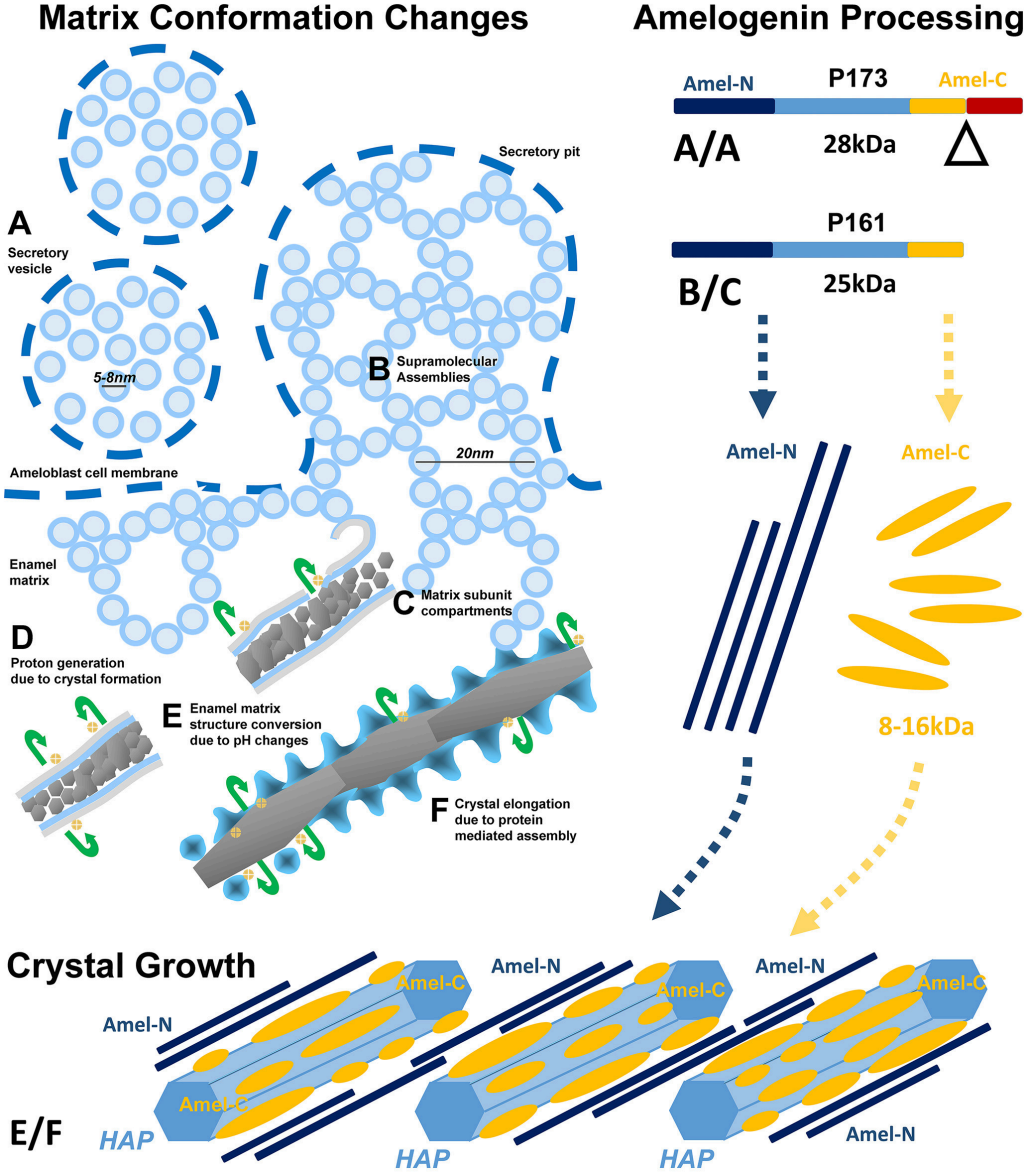
Model explaining enamel crystal formation through matrix assembly and processing. **(A–F)** Changes in matrix conformation. Enamel matrix assembly begins as 5–7 nm subunits within ameloblast secretory vesicles **(A)**. Once secreted into the extracellular space, mineral-rich enamel proteins self-assemble as 20 nm diameter subunit compartments that provide the structural basis for orderly spaced enamel crystal nucleation **(B,C)**. Proton generation during initial crystallization results in a dissociation of the stippled materials matrix and a “shedding” of enamel protein assemblies onto the surfaces of growing enamel hydroxyapatite crystals **(E,F)**. **(A/A–B/C)** Temporo-spatial amelogenin processing during enamel maturation. (A/A) Full-length P173 amelogenins are exclusive to the enamel organ (Figure 4C lane 1), where they are packaged into 5–8 nm subunits within secretory vesicles (Figures 1A,B, 2A,B). Upon entry into the enamel extracellular matrix, cleavage of the hydrophilic C-terminus generates P161 amelogenins (Figure 4C lanes 2,3), and resulting hydrophobic interactions between P161 amelogenins trigger the formation of 20 nm sized subunit compartments (“nanospheres,” Figures 1C, 2C) for the spacing of enamel crystal nucleation sites. (E/F) N- and C-terminal amelogenins during enamel crystal formation and elongation. Further processed amelogenin C-terminal fragments (Figure 4C lane 6, 8–16 kDa) are tightly associated with the elongating crystal surface (Figures 1E, 2D,E) as revealed by guanidine extracts. In contrast, N-terminal amelogenins likely float in between elongating apatite crystals as they were only detected in SDS detergent extracts and not in the guanidine fraction (Figure 4D).

In conclusion, our *in vivo*, organ culture, and amelogenin *in vitro* assembly studies have resulted in a dynamic three-phase model of enamel matrix transformation and crystal growth (Figure 5). Based on our data and other findings presented in this contribution, enamel matrix assembly begins as 5–10 nm subunits formed by full-length amelogenins within ameloblast secretory vesicles (A). Once secreted into the extracellular space, mineral-enriched enamel protein self-assemblies consisting of C-terminally cleaved amelogenins organize into 20–25 nm diameter subunit compartments that provide the structural basis for orderly spaced enamel crystal nucleation (B,C). Proton generation during initial crystallization results in further matrix reorganization and amelogenin processing, a dissociation of the stippled materials matrix and a “shedding” of C-terminal amelogenin/mineral nanoclusters onto the surfaces of growing enamel hydroxyapatite crystals (E,F).

## Ethics statement

All animals studies were approved by the Institutional Animal Care Committee of the University of Illinois at Chicago.

## Author contributions

MP and TD wrote the article, TD designed the experiments, TL, LL, MA, TJ, and XL conducted experiments.

### Conflict of interest statement

The authors declare that the research was conducted in the absence of any commercial or financial relationships that could be construed as a potential conflict of interest.
